# Rheumatismus, or Rheumatism

**Published:** 1838-05-09

**Authors:** N. Chapman

**Affiliations:** Professor of the Theory and Practice of Physic, in the University of Pennsylvania


THE MEDICAL EXAMINER.

DEVOTED TO MEDICINE, SURGERY, AND THE COLLATERAL SCIENCES.

EDITED BY J. B. BIDDLE, M. D. AND M. CLYMER, M. D.

No. 10.] PHILADELPHIA, WEDNESDAY, MAY 9, 1838. [Vol. I.

### RHEUMATISMUS, OR RHEUMATISM.

*By* N. Chapman, M. D., *Professor of the Theory and Practice of Physic, in the University of Pennsylvania.*

(Concluded from page 140.)

Of the treatment of acute inflammatory rheumatism, much of what I should otherwise have had to say, has been anticipated, in that of gout. As to the disease in our own climate, at least, it will be right to commence the cure with copious venesection, which is pointedly called for, by the symptoms, and by the appearance of the blood, this being, perhaps, more inflammatory, than in any other affection. No substitute exists for the lancet in this case. The other remedies will be unproductive of utility, and never should be prescribed, till the intensity of action is overcome by direct depletion. It is my wish, to press this precept upon you strongly, since in most of the writers, you will find bleeding cautiously advised, and by some altogether interdicted, as nugatory in relation to the cure, calculated to lead to sudden translation of the disease to vital parts, or to run into the chronic state. These are the objections of theory or ignorance, and must not be listened to, for an instant. Bleeding here, when adequately urged, is nearly as effectual in arresting the attack, as in the other phlegmasiæ, and, instead of promoting, has a contrary effect, as to the alleged metastasis. The instances of the kind, amounting to several, which have come under my notice, occurred where no blood had been lost. It is an error to suppose, that disease is invited to a part by the weakness of it, which seems to be the foundation of the dread of the remedy. The reverse is true, or it is attracted by a pre-existing phlogistic irritation, endowing it with an increased susceptibility, by the removal of which, and bleeding is the best means of doing it, the chance of its happening is diminished. By this mode, a sprain or other injury attracts it, and do we not find the same effect from irritating revulsives, as rubefacients, sinapisms, or blisters, exemplified, especially, in the operation of these applications in retrocedent or misplaced gout. Not less absurd is the supposition, that bleeding promotes the transmutation of the disease into the chronic state. It is a rule, to which rheumatism forms no exception, that, proportioned to the rapidity with which acute inflammations are reduced, and to this end, surely, the loss of blood is most efficient, is the security against such a result. Nor is any thing more certain to my mind, than that the tendency in the disease which has also been complained of by some, to cerebral disturbance and petechial eruptions, must be ascribed to the premature substitution of a stimulating and tonic, for the antiphlogistic course, and, especially, venesection.

But very differently is the process appreciated by the writers of nearly every country of Europe. To so great an extent has this prejudice against bleeding been carried, by some of the British practitioners, especially, that even the Peruvian bark was substituted in its place! During my attendance in the London hospitals, the use of it was generally adopted, and, undoubtedly, it has received the sanction of several of the highest English authorities, for more than a century. By Morton, the contemporary of Sydenham, it was probably introduced, and claims, subsequently, the distinguished support of Heberden, Fothergill, Saunders, Baker, Fordyce, Haygarth, &c. It is really extraordinary to see the confidence with which the bark is spoken of by Haygarth, who, on the most ample experience with it, says: "Except mercury, in syphilis, there are few, and, perhaps, no examples, where a remedy produces such speedy relief, and perfect recovery, in so formidable a disease. For many years, I have been thoroughly convinced that the Peruvian bark has a much more powerful effect in the rheumatic, than in any other fever, and that it does not cure even an ague, so certainly, or so quickly."

Nor have the European practitioners become emancipated entirely from the thraldom of this error, even at the present moment. Elliotson and Johnson, two of the most conspicuous of the English, at least, seem to labor under it, and we are told that it universally continues with those of France.

That there is a very egregious fallacy in these statements, is obvious. Never did I perceive the least advantage, in any one case of the early stage of the disease, from the practice, and often, the most manifest mischief, in which sentiment, I am fully supported by the best authorities of our country. Yet, I shall presently show you, that, properly applied, the bark is valuable in this disease.

Nor is it to be supposed, from what I have said, that all the British writers concur in the proscription of bleeding, in rheumatism. This is very far from the fact, since, among others, it is strenuously recommended by Sydenham, Pringle, Cullen, Scudamore, &c.

It is very amusing to us, who have been so long and so thoroughly conversant with this practice, to listen to the ignorance or presumption of a late French writer, in preferring his pretensions to it as a discovery of his own. Bouillaud, whom I have previously cited, in a late publication, after harshly censuring Louis, Chomel, and others of his contemporaries, who, he says, pursue an opposite method, proclaims, in a burst of enthusiasm, the utility of blood-letting in the disease, as a revelation, for the first time, made to himself alone!

Next, the bowels ought to be freely evacuated, and the saline articles are mostly preferred for the purpose. But the combination of calcined magnesia, Epsom salts, and the tincture of colchicum, noticed formerly, is, according to my own experience, peculiarly well suited. It evacuates the bowels, adequately, and controls more effectually the rheumatic action. Yet purging by any article is here less serviceable than in the kindred arthritic affection, probably from rheumatism's not, like gout, primarily originating in a depravation of the alimentary canal. My remarks have reference to ordinary rheumatism. The case being modified by miasmatic influence, then, purging, and especially by calomel, proves exceedingly efficacious, and under all circumstances does good, on the principle of reducing vascular action, and febrile excitement, as well as by preparing the way for the efficient operation of other remedies.

Emetics, have also been directed in rheumatism. Excepting in the form of the disease justindicated, I have no knowledge of them, and must therefore, advance an opinion diffidently, in relation to their powers. The stomach is here much oppressed, and nauseated, and they prove serviceable in a mode, which I need not explain.

As somewhat confirmatory of their efficacy, the fact may be stated, that in this variety of the disease, whenever spontaneous vomiting takes place, there is for a time, a very marked mitigation of both the general and local affections.

Evacuations having been thus premised, the treatment, whatever may be the precise character of the case, is resolved into the use of the febrifuges. Combined in small portions with the nitrate of potash, and sometimes calomel, the tartarised antimony is greatly prescribed. By this preparation, vascular action is further subdued, or kept under, and, what is equally important, the skin may be restored to a more healthy condition.

It is at this conjunction, or even at an earlier period, that the contra-stimulant plan of the Italian school, consisting of immense doses of emetic tartar, is proposed. What was said of it in relation to pneumonic inflammation, is equally pertinent to the present disease, and I must be content to refer to the criticism on the practice delivered on that occasion.

Febrile excitement becoming further reduced, a recurrence is usually had to the more direct means of sweating. This is a mode of combating rheumatism, which has long been consecrated to the purpose, by the undivided opinions of every description of practitioners, and is too often abused. It is well ascertained, that sweating is always pernicious, if practised in the early or phlogistic stage. Even when it comes on spontaneously, it never, as previously stated, affords any relief, and usually aggravates the distress. We commence with the milder diaphoretics, and, afterwards, employ articles rather more stimulating, of which the most efficacious is the Dover's powder. But it is a popular practice of our country, not without some professional sanction, to treat this state of rheumatism, with the Virginia snakeroot, the thoroughwort, and, above all, by the pleurisy-root. Each of these articles being very certainly diaphoretic, I have no doubt, when judiciously directed, of their utility.

To the prohibition of early and active sweating, an exception exists in the torpid or tetanoid variety of the disease. The indication here, at first, is to awaken sensibility, and to bring on a reaction, with which intentions, I have found the vapour bath and frictions, aided by the Dover's powder, perseveringly continued, the most effectual. Development of action having taken place, venesection, and its auxiliaries may be resorted to with those other means appropriated to the more ordinary forms of the disease, in the several stages.

As in gout, diuretics sometimes prove of immense advantage in rheumatism. Nearly the same articles, as well as the rule for their employment, are suited to both cases.

Colchicum, in union with the spirits of nitre, is especially worthy of trial. Its properties are very peculiar, and it seems, on some occasions, to display a sort of specific agency. But the pipsissewa, pyrola umbellata, has acquired so much repute, as to have had bestowed on it the popular title of *rheumatism weed*, and may hence have claims to attention.

These various remedies failing, and where, especially, evidence arises of a disposition in the case to glide into the chronic state, we should not hesitate to interpose mercury, with a view to a moderate impression, which usually proves a very decisive process. Having presently occasion to say more of it, I shall now only further remark, that, under the circumstances immediately before us, it will be found often expedient, and, particularly, if much pain exists, to unite opium, and, sometimes, ipecacuanha, to the calomel.

In attending to these general indications, the local affection is not to be overlooked. The pain of it, when violent, can hardly be borne, and has, certainly, the effect of keeping up fever. On every account, therefore, we should endeavour to do it away. But, as in gout, some difference of opinion prevails, as to the proper measures to be adopted. Cold applications, even snow or ice, have been lately employed. But I cannot be persuaded of their general propriety. Granting for a moment, the utility of them, I should much apprehend a metastasis. Yet I am in possession of some evidence in support of this practice, of too much importance to be withheld. By Professor Jackson of Boston, I have understood, it is constantly pursued, and not less by the physicians of Russia, and Spain, and, as is represented, with unequivocal advantage. Yet I repeat, that my own experience, confirmed by the tenor of medical authority, is against it, for the reason assigned.

No great relief is, however, afforded by opposite means or warm fomentations, though I cannot allow that they are utterly destitute of effect. The hop poultice I have found the best of such applications. It is said, however, that steaming the part by means of an apparatus lately contrived, is still more lenitive and emollient. By Scudamore his mixture is strongly recommended, the composition of which has been noticed. Cabbage leaves are sometimes palliative, and still more so, are those of the tulip-poplar, placed on the part.

In the last few years, the practice of Mr. Balfour has attracted no inconsiderable attention. It consists in bandaging, as tightly as can be endured, the affected part by a flannel roller, with the occasional use of frictions. Whatever may be its utility, in some other applications, of which I am hereafter to speak, I cannot consider it adapted to an inflamed and sensible condition of the local affection.

Discarding most of the preceding measures as nugatory, or of inferior efficacy, our main reliance is to be placed on topical bleeding by leeches or cups, frequently renewed, and next on repeated blisters. Governed by those theoretical notions, to which reference was formerly made, these appliances are now directed by some practitioners to the spine. That, on certain occasions, they have thus answered better, I am unwilling to deny, though, at the same time, I must insist, that, where more successful, the cases were not genuine rheumatism. By revulsion, bleeding from a distant point may be doubtless serviceable. Yet it is equally true, that it oftener fails, and is generally less effectual than when practised near the affected part.

In regard to the use of opium, we are governed, very much, as in gout. It seldom fails, according to my experience, to exasperate *acute*, *unsubdued* rheumatism. Even in the form of Dover's powder, and where it produces perspiration too, it mostly, without abating pain, adds to the heat and restlessness. This fact is particularly entitled to attention, as patients, in extreme anguish, very often demand, in a clamorous manner, a dose of the medicine.

Though the practice, with opium, should be generally regulated by the preceding cautions, there are cases, in which it may be resorted to, at a much earlier period. The form of the disease, to which I refer, sometimes succeeds genuine rheumatism, after a few days continuance. It is, however, more commonly met with as an original state in women, or other persons of weak and irritable habits. There is, here, no force of vascular action, and incomparably more of irritation, than inflammation. Either alone, or in conjunction with calomel and ipecacuanha, as has before been mentioned, opium is unequivocally serviceable.

Located in the more diffused portions of the fibrous tissue, and, especially, the periosteum, essentially the same course is to be pursued in the disease. What I shall say of the affection of the pericranium, to which my remarks will be confined, may suffice as a general direction. The remedies, here, are steaming, leeching, a blister to the nape of the neck, and keeeping the head warm by an envelope of cotton, or wool, or a flannel or fur cap. Combined with these topical means, evacuants of the bowels are serviceable, nauseants are so, also, and vomiting will promptly relieve it, on some occasions. As further means, opiate diaphoretics are deserving of confidence. Many persons, with thin hair, or entirely bald, are very susceptible of such attacks, on the slightest exposure. Wearing a wig by day, and a warm cap at night, I have hardly ever known to fail as preventives.

---

At a commencement held Friday, April 6th, 1838, the degree of Doctor of Medicine, in the University of Pennsylvania, was conferred upon an hundred and forty-four graduates. Thirteen had received the degree in July, 1837, making, in all, one hundred and fifty-seven.

---

M. Heurteloup, by request of the emperor of Russia, is about to write a treatise on Lithotripsy.

